# Gene Regulatory Interactions at Lamina-Associated Domains

**DOI:** 10.3390/genes14020334

**Published:** 2023-01-28

**Authors:** Julia Madsen-Østerbye, Mohamed Abdelhalim, Sarah Hazell Pickering, Philippe Collas

**Affiliations:** 1 Department of Molecular Medicine, Institute of Basic Medical Sciences, Faculty of Medicine, University of Oslo, 0317 Oslo, Norway; 2 Department of Immunology and Transfusion Medicine, Oslo University Hospital, 0424 Oslo, Norway

**Keywords:** adipose stem cell, adipogenesis, chromatin loop, enhancer interaction, lamina-associated domain, nuclear lamina

## Abstract

The nuclear lamina provides a repressive chromatin environment at the nuclear periphery. However, whereas most genes in lamina-associated domains (LADs) are inactive, over ten percent reside in local euchromatic contexts and are expressed. How these genes are regulated and whether they are able to interact with regulatory elements remain unclear. Here, we integrate publicly available enhancer-capture Hi-C data with our own chromatin state and transcriptomic datasets to show that inferred enhancers of active genes in LADs are able to form connections with other enhancers within LADs and outside LADs. Fluorescence in situ hybridization analyses show proximity changes between differentially expressed genes in LADs and distant enhancers upon the induction of adipogenic differentiation. We also provide evidence of involvement of lamin A/C, but not lamin B1, in repressing genes at the border of an in-LAD active region within a topological domain. Our data favor a model where the spatial topology of chromatin at the nuclear lamina is compatible with gene expression in this dynamic nuclear compartment.

## 1. Introduction

Adequate control of gene expression at the periphery of the nucleus is critical for the proper regulation of differentiation [1]. At the nuclear periphery, chromatin interacts with the nuclear lamina (NL), a meshwork of A- and B-type lamins (lamins A/C, B1 and B2), via 10-kb to 10-mb lamina-associated domains (LADs) [2,3]. LADs are enriched in di- and tri-methylated H3K9 and contain a low density of mostly repressed genes. Whereas most LADs are conserved between cell types (these are often called constitutive LADs or cLADs), some are more variable (facultative LADs or variable LADs; vLADs) and differ between cell types [4,5] through repositioning during differentiation [6,7,8,9,10]. This dynamics, together with LAD alterations reported in laminopathies [11,12,13,14], cancer and senescence [15], underlines the functional relevance of these genome organizers.

Not all LADs are uniform in their chromatin composition. About 10% of genes in LADs are expressed and escape the repressive environment of the NL by being in euchromatic regions within LADs [16,17,18]. We have reported, within cLADs, active regions containing differentially expressed (DE) genes during early stages of the differentiation of human adipose stem cells (ASCs) into adipocytes [10]. In line with other findings [16,17,19], these regions are depleted of H3K9me3 and show lower LMNB1 enrichment than the rest of the LAD. How DE genes in LADs are regulated is largely unknown.

Association of multiple enhancers with genes is a safeguard mechanism of gene expression control [20]. Recent enhancer-capture Hi-C (ECHi-C) data show that enhancers exist as intra-connected “communities” whose remodeling via gains and losses of contacts upon differentiation contributes to defining lineage specificity [21]. Moreover, enhancers, genes or entire gene units are released from the NL during neuronal [6], myogenic [22] or adipogenic [7,9,10] differentiation, or after T cell activation [8,23], providing one mechanism of cell type-specific gene expression regulation at the nuclear periphery. 

Chromatin at the nuclear periphery also harbors enhancers between LADs [24]. It is however still unclear whether genes at the NL are able contact regulatory sites within or outside LADs. Enhancers imputed to genes in LADs have been proposed to engage in short-range interactions within LADs [10], whereas others seem to lack NL association, suggesting more long-range interactions [16]. Here, we integrate published ECHi-C data for mesenchymal stem cells [21] with our own transcriptome, chromatin state and microscopy data and manipulate lamin levels to show that expressed genes in cLADs are able to connect to enhancers in LADs and inter-LADs. Our data favor a view where chromatin conformation at the NL is compatible with gene expression in this nuclear compartment. 

## 2. Materials and Methods

### 2.1. Adipose Stem Cell Culture and Adipogenic Induction

ASCs were isolated from subcutaneous liposuction material and cultured as described [25]. In short, ASCs were allowed to proliferate in DMEM/F12 medium containing 10% fetal calf serum and 20 ng/mL basic fibroblast growth factor. Cells at passages 4–7 were used. For adipogenic differentiation, proliferating ASCs were harvested and reseeded at confluency in DMEM/12/fetal calf serum as above but without the basic fibroblast growth factor. Cells were cultured in this medium in a confluent state for 48 h and induced to differentiate on day 0 (D0) by the addition of an adipogenic cocktail (10 µg/mL insulin, 200 µM indomethacin, 1 µM dexamethasone and 0.5 µM 3-isobutyl-1-methylxanthine). Differentiation medium was renewed every 3 days. Cells were harvested for analyses before induction of differentiation (D0) and on D1, D3, D6 and D9. 

### 2.2. Immunoblotting

Proteins were separated by a 4–20% gradient SDS-PAGE (for LMNA/C, LMNB1 and γ-Tubulin) and transferred to an Immobilon-FL membrane (Millipore). Membranes were blocked with Odyssey blocking buffer (LI-COR) (LMNB1), 5% BSA (LMNA/C) or 5% milk (γ-Tubulin) and incubated with antibodies to LMNB1 (1:1000; Abcam ab16048), LMNA/C (1:1000 Santa Cruz sc7292x) or γ-Tubulin (1:10,000; Sigma-Aldrich Norway; Oslo, Norway; T5326). Proteins were detected with IRDYE-800-coupled antibodies or Peroxidase-conjugated antibodies. Relative protein levels were quantified using Image Lab (Bio-Rad Laboratories; Hercules, CA, USA)). 

### 2.3. Fluorescence In Situ Hybridization (FISH)

Fosmid FISH probes were purchased from BACPAC Genomics (Emeryville, CA, USA) (bacpacresources.org; accessed on 16 January 2023). Probes were designed based on RNA expression, ChIP-seq profiles of histone modifications and enhancer identification [10] (Table S1). FISH was done as described [26]. In short, cells were swollen in hypotonic buffer, fixed in ice-cold methanol/acetic acid and dropped on slides. Probes covering genes and enhancers were respectively labeled with digoxigenin-11-dUTP and Biotin-16-dUTP. Per slide, 80 ng probe was mixed with 8 µg *Cot*-1 DNA and 10 µg salmon sperm DNA, precipitated and DNA dissolved in 12 µL hybridization mix. Slides were RNase-treated and washed in 2x SSC, dehydrated in ethanol, air-dried, denatured for 1 min 20 s at 70 °C in 70% deionized formamide/2x SSC, dehydrated and air-dried again. Probes were denatured at 70 °C, pre-annealed for 15 min at 37 °C and 15 µL was applied onto 22 × 22 mm coverslips, which were then mounted on slides with nuclei. 

Slides were hybridized overnight at 37 °C and washed in 2× SSC (45 °C for 4 × 3 min) and in 0.1× SSC (60 °C for 4 × 3 min). Slides were blocked in 5% milk in 4× SSC for 5 min and incubated with anti-digoxigenin (mouse, 0.4 µg/mL; Roche, Oslo, Norway). Slides were washed in 4× SSC/ 0.1% Tween-20 for 3 × 2 min and incubated with Avidin Alexa Fluor 488 (rabbit, 1.7 µg/mL; Invitrogen, Waltham, MA, USA) and anti-mouse Alexa Fluor 594 (rabbit, 2.5 µg/mL; Jackson ImmunoResearch; Ely, UK). Slides were washed and incubated with anti-rabbit Alexa Flour 594 (donkey, 2.5 µg/mL; Jackson ImmunoResearch) and biotinylated anti-Avidin D conjugate (goat, 1.0 µg/mL; Vector Laboratories; Burlingame, CA, USA). Slides were washed and incubated with Avidin Alexa Fluor 488 (rabbit, 1.7 µg/mL; Invitrogen), washed and mounted with 0.2 µg/mL DAPI in mounting medium. Images were taken in DeltaVision (dv) image stack format. Fifty FISH images of nuclei containing two green and two red FISH signals (100 alleles) were analyzed using NEMO [27], where FISH probe-probe distance and FISH probe distance to the nuclear periphery delineated by DAPI were measured. 

### 2.4. Knock-Down of LMNA/C and LMNB1

ASCs were plated at 50% confluency in proliferation medium. After 24 h, knock-down of LMNA/C, LMNB1 or both LMNA/C and LMNB1 was done using Lipofectamin RNAiMAX (Thermo Fisher Scientific, Oslo, Norway) according to manufacturer’s recommendations using siLMNA/C:siRNA IDS8221, siLMNB1:siRNA IDS8224 or siScr:siRNA ID4390844 as the control (Ambion; Thermo Fisher Scientific) at final concentrations 40 nM. After 24 h, the media was replaced, and cells kept proliferating for five days. Cells were reseeded, plated at confluency and differentiated as described in Section 2.1.

### 2.5. Chromatin Immunoprecipitation (ChIP)

ChIP of LMNA/C, H3K4me3, H3K4me1, H3K27ac, H3K9me3, H3K27me3 and H3K36me3 was done as described [7]. Briefly, cells were fixed with 1% formaldehyde, lysed in 50 mM Tris-HCl, pH 8, 10 mM EDTA, 1% SDS, protease inhibitors and Na-butyrate, and sonicated into ~200 bp fragments using a Picorupter (Diagenode; Seraing, Belgium). After sedimentation, the chromatin supernatant was incubated with anti-LMNA/C (Santa Cruz sc7292x), H3K27ac (Diagenode c15410174), H3K4me3 (Diagenode c15410003), H3K4me1 (Diagenode c15410037), H3K9me3 (Diagenode C15410056), H3K27me3 (Diagenode C15410069) or H3K36me3 (Diagenode C15410058) antibodies, each at 2.5 µg/10^6^ cells (histones) or at 10 µg/5 × 10^6^ cells (LMNA/C). Cross-links were reversed and DNA was purified. ChIP libraries were prepared using a Microplex kit (Diagenode) and sequenced on a Nextseq 500 or Novaseq (Illumina; San Diego, CA, USA). 

### 2.6. ChIP-Seq Analysis and Chromatin State Modeling

LMNB1 ChIP-seq data generated by us [28] were downloaded from NCBI Gene Expression Omnibus (GEO) GSE109924. LMNA/C ChIP-seq was done on D0, D1 and D3 of two independent adipose differentiations. Reads were aligned to hg38 after removing duplicates using Picard MarkDuplicates (http://broadinstitute.github.io/picard/; accessed on 16 January 2023) and data processed as described [10]. Each pair of mapped ChIP and input read files contained the same read depth by down-sampling reads for each chromosome. Mean LMNA/C Log2(ChIP/Input) was calculated using WiggleTools v1.2 (https://github.com/Ensembl/WiggleTools; accessed on 16 January 2023) [29]. Bigwig tracks were generated from Log2(ChIP/Input) ratios in 1 kilobase (kb) bins using bamCompare from Deeptools v3.5.1 (https://github.com/deeptools/deepTools/releases/tag/3.5.1; accessed on 16 January 2023) [30]. 

From our H3K4me1, H3K4me3 and H3K27ac ChIP-seq data, accessed from GEO GSE185066 [10], Bigwig files for normalized read counts were used to generate enrichment profiles ±4 kb around the transcription start site (TSS) using Deeptools v3.5.1 [30]. For histone ChIP-seq data generated for this study, reads were mapped to hg38 using Bowtie2 v2.4.1 (https://github.com/BenLangmead/bowtie2; accessed on 16 January 2023) [31]. Duplicates were removed along with multi-mapping reads and alignments with a mapping quality below 10. Peaks were detected using MACS2 v2.2.7.1 (https://github.com/macs3-project/MACS/releases/tag/v2.2.7.1; accessed on 16 January 2023) [32]. 

ChromHMM [33] was used to identify chromatin states using aligned reads. Options were selected to learn a 15-state model using the Baum-Welch training algorithm [34] on the grounds that it represented non-redundant states concordant with genomic locations. Fold-enrichment of ECHi-C targets was calculated as follows: ((No. bases in state and external annotation)/(No. bases in state))/((No. bases in the external annotation)/(No. bases in the genome)) [33]. 

Genomic data were viewed in the Integrative Genomic Browser (IGV) (www.igv.org; accessed on 16 January 2023) [35] or in the University of California Santa Cruz (UCSC) Genome Browser (https://genome.ucsc.edu/; accessed on 16 January 2023) [36], as indicated. 

### 2.7. ATAC-Seq Data Reprocessing

Assay for Transposase-Accessible Chromatin (ATAC)-seq data were downloaded from GEO GSE143450 (https://www.ncbi.nlm.nih.gov/geo/query/acc.cgi?acc=GSE143450; accessed on 19 January 2023) [37]. Coverage tracks were lifted from hg19 to hg38 for samples from gluteofemoral fat. Both ATAC-seq replicates from each of 10 donors in that study were merged, and mean coverage was used to generate an ATAC-seq profile at the TSS. 

### 2.8. Intersections between LADs, Genes, and Enhancer Positions

Published ECHi-C data, acquired during the differentiation of human mesenchymal stem cells into adipocytes by Madsen et al. [21], were downloaded from GEO GSE140782. Both bait and target coordinates were lifted from hg19 to hg38, and significant connections (*p* ≤ 0.01; [21]) from all time-points confounded in that study were used. Intersects between baits, targets, genes and histone marks were determined using BEDTools v2.29.2 (https://github.com/arq5x/bedtools2; accessed on 16 January 2023) [38] and BEDOPS v2.4.37 (https://github.com/bedops/bedops/releases; accessed on 16 January 2023) [39]. Intersects between targets and LADs were determined using Intervene v0.6.4 [40]. All intersects required at least 1 base-pair (bp) overlap between features.

### 2.9. Gene Expression Analysis

The RNA-seq data used here were published by us [10] and downloaded from GEO GSE 185066. An expressed gene was defined by a transcript normalized read count of ≥15 [10]. For reverse transcription (RT)-qPCR, RNA was isolated using the RNAeasy mini kit (Qiagen). In addition, cDNA was synthesized with high-capacity cDNA reverse transcription kit (Applied Biosystems), and qPCR was done using IQ SYBR green (Biorad) with *SF3A1* as reference gene and using primers listed in Table S2. Gene Ontology was analyzed using Protein ANalysis THrough Evolutionary Relationships (PANTHER) (http://www.pantherdb.org/; accessed on 16 January 2023) [41].

## 3. Results

### 3.1. cLADs Contain Active Genes in Euchromatic Regions of Low Enrichment in A- and B-Type Lamins

**Figure 1:**
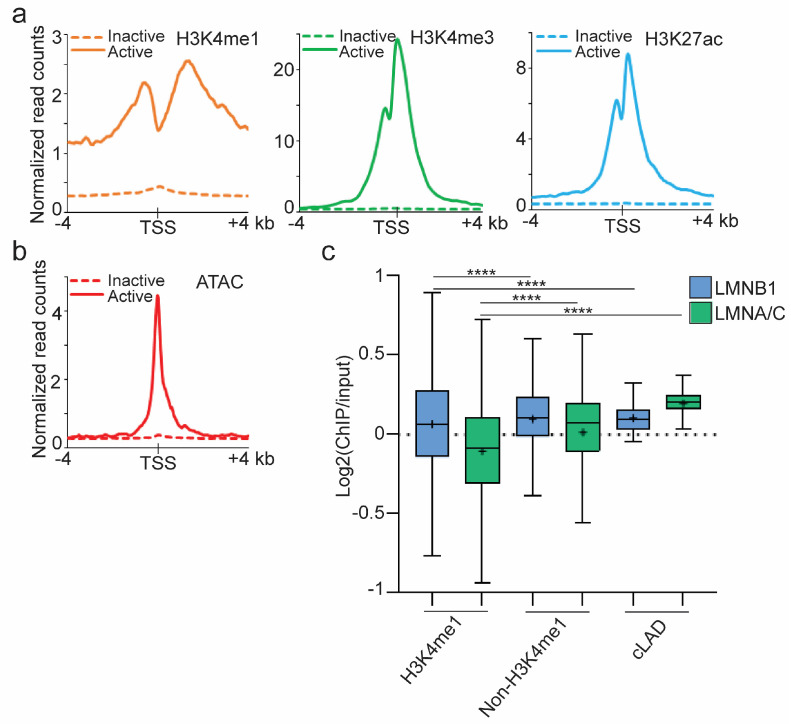
Adipogenic cLADs contain active gene regions. (**a**) H3K4me1, H3K4me3 and H3K27ac levels around the TSS of expressed (n = 244) and non-expressed (n = 3702) genes in cLADs; mean ChIP-seq read counts normalized to library size. (**b**) Chromatin accessibility around the TSS of cLAD genes, determined from published ATAC-seq data [37]. (**c**) LMNB1 and LMNA/C enrichment in H3K4me1 and non-H3K4me1 cLAD regions, and in whole cLADs; bar, median; cross, mean; box, 25–75% percentile; whiskers, min-max **** *p* < 10^−4^, unpaired two-tailed *t*-test with Welch’s correction. A H3K4me1 region here is a cLAD region containing ≥ 1 H3K4me1 peak; LMNB1 and H3K4me1 data are from [10].

We recently reported transcriptionally active regions in LADs common to undifferentiated ASCs and early adipocytes (cLAD) as determinants of LAD repositioning in the first 3 days of adipogenic differentiation of primary human ASCs [10]. These regions are delineated by H3K4me1 enrichment (H3K4me1^high^), low in LMNB1 (LMNB1^low^) and depleted of H3K9me3 [10]. Extending these findings in the same adipogenic differentiation system, we now report 244 expressed genes in adipogenic cLADs (Table S3). Gene ontology analysis reveals implications in cell adhesion, signaling and developmental processes, which are typical for genes in LADs [5,10] (Table S4). Using our previous histone ChIP-seq data [10], we find that expressed cLAD genes are enriched in H3K4me3, H3K4me1 and H3K27ac around the TSS (Figure 1a). Further, by integrating recent ATAC-seq data for human adipose tissue from several donors [37], we show that the TSS of expressed cLAD genes also reside at sites of open, accessible chromatin (Figure 1b). 

The NL consists of networks of A- and B-type lamins [42], which can interact with chromatin through shared and distinct LADs [43]. We established, by ChIP-seq of LMNA/C (Figure S1a), that LMNB1 cLADs we previously mapped during early adipogenic differentiation [10] were co-enriched in LMNA/C (Figure S1b), and that H3K4me1^high^ cLAD regions, which are LMNB1^low^, were also low in LMNA/C compared to the rest of the LADs (*p* < 10^−^^4^, unpaired two-tailed *t*-test with Welch’s correction; Figure 1c). Thus, euchromatic active cLAD regions showed low contact frequencies with both A- and B-type lamins, suggesting that they were locally detached from the NL. 

### 3.2. ECHi-C Data Provide Evidence of Enhancer Connectivity at the Nuclear Periphery

**Figure 2:**
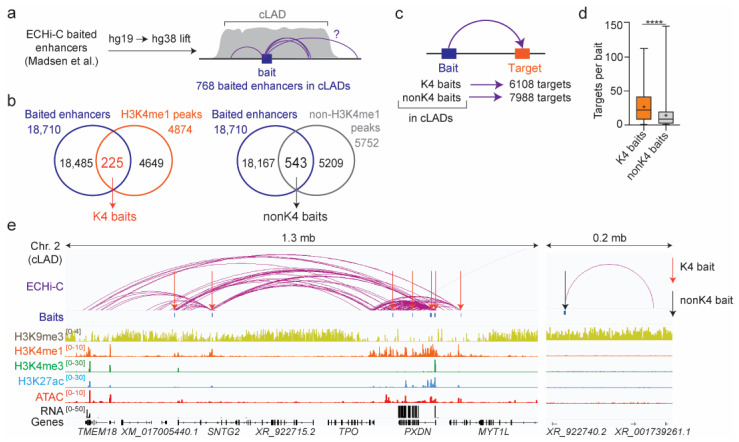
Chromatin interactions at the nuclear lamina. (**a**) Numbers of ECHi-C baited enhancers from Madsen et al. [21] (baits) mapped to the cLADs of our study. (**b**) Baited enhancers intersecting with H3K4me1 peaks (K4 baits) or non-H3K4me1 peaks (nonK4 baits) in cLADs. (**c**) Numbers of ECHi-C targets of cLAD K4 baits and nonK4 baits. (**d**) Numbers of ECHi-C targets per K4 and nonK4 bait; bar, median; cross, mean; box, 25–75% percentile; whiskers, min-max; **** *p* < 10^−4^; unpaired *t*-test with Welch’s correction. (**e**) Genome browser view of ECHi-C interactions originating from K4 baits (red arrows) and from a nonK4 bait (black arrow) in a cLAD, with underlying histone modifications and ATAC sites. Gene expression is shown (RNA). Histone ChIP-seq and RNA-seq data are from [10]; ATAC-seq data are reprocessed from [37].

Whereas transcription can elicit the local dissociation of chromatin from the NL [18,44], chromatin looping involving CTCF and cohesin has been shown to also mediate detachment from the NL [19]. Thus, we examined whether active cLAD regions were involved in chromatin interactions which could account for the expression of cLAD genes. To this end, we took advantage of recent ECHi-C data for human mesenchymal stem cells induced to differentiate into adipocytes [21]. After lifting interaction bed files from the hg19 to hg38 genome assembly, we pooled all ECHi-C baited enhancers (“baits”) and corresponding significant targets (defining enhancer connections) for all differentiation time-points confounded in that study. By intersecting the genomic coordinates of these 18,710 baits with the cLAD coverage of our study (through D0, D1 and D3 of differentiation), we identified 768 baits localized within our adipogenic cLADs (Figure 2a). 

Next, we cataloged baits overlapping by ≥ 1 bp with H3K4me1 peaks in cLADs. We focused on this subset of baits because coincidence with H3K4me1 would also confirm an enhancer marking in our adipogenic system, and H3K4me1^high^ regions demarcate H3K9me3-depleted and LMNB1^low^ cLAD regions containing active genes [10] (see Figure 1d). We found 225 such H3K4me1-marked baits, henceforth called “K4 baits,” and 543 baits not overlapping with any H3K4me1 peak in cLADs (“nonK4 baits;” Figure 2b). 

From the ECHi-C dataset, we then identified 6108 targets (connections) from the K4 baits and 7988 targets from the nonK4 baits (Figure 2c), highlighting a higher density of connections from K4 baits (mean: 27.5/bait) than from nonK4 baits (mean: 14.7/bait) within cLADs (Figure 2d). Although this was anticipated, this finding argues that active genomic regions at the NL are able to contact other parts of the genome. This is exemplified in a > 1 mb cLAD region on chromosome 2, which displayed several K4 baits connecting weak (H3K4me1) or active (H3K4me1/H3K27ac) enhancers within the *PXDN* locus or between *PXDN* and *SNTG2* or *TMEM18*, ~1 mb upstream (Figure 2e). We also noted a nonK4 bait within an H3K9me3 segment of the same LAD, connecting two unannotated loci 140 kb apart (Figure 2e). Contact density was extremely low from this nonK4 bait, supporting our quantification (Figure 2d) and reflecting the overall compact and inaccessible state of H3K9me3 chromatin (Figure 2e). 

**Figure 3:**
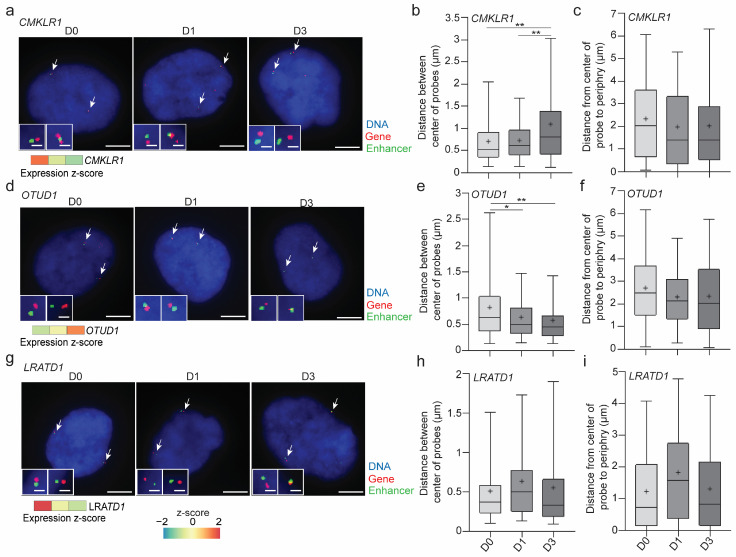
FISH analysis of gene-enhancer distances within LADs during early adipogenic differentiation. (**a**–**c**) *CMKLR1* locus: (**a**) FISH images of changes in proximity of the *CMKLR1* gene (red probe) and a putative (GeneHancer-predicted and ECHi-C-predicted) enhancer 480 kb away (green probe) on D0, D1 and D3 of differentiation; blue, DAPI; scale bars, 5 µm in main, 0.5 µm in insets; a z-score RNA-seq heatmap is shown for D0, D1, D3 (left to right), adapted from [10]; (**b**) probe-probe distances; ** *p* = 0.04; unpaired two-tailed *t*-test; (**c**) distances from probe to nuclear periphery outlined by DAPI staining (green probes). (**d**–**f**) FISH analysis of the *OTUD1* locus and a putative enhancer 348 kb away, as in (**a**–**c**); * *p* = 0.08, ** *p* = 0.04; unpaired two-tailed *t*-test. (**g**–**i**) FISH analysis of the *LRATD1* locus and a putative enhancer 510 kb away, as in (**a**–**c**). For all loci, n = 50 nuclei each with two green and two red FISH signals; box plots: bar, median; cross, mean; box, 25–75% percentile; whiskers, min-max.

To appreciate the active gene-enhancer proximity in cLADs at the cellular level, we analyzed gene-enhancer distances on D0, D1 and D3 of adipogenic differentiation by dual-color FISH labeling an expressed LAD gene and an enhancer located >200 kb from the gene probe position, marked by H3K27ac in our dataset (Table S1). We found that the *CMKLR1* locus displayed increasing gene-enhancer distances as *CMKLR1* was downregulated in this time course (Figure 3a,b). This occurred at the nuclear periphery (≤2 µm from the DAPI border), suggesting chromatin repositioning in the vicinity of the NL (Figure 3c). In contrast, the *OTUD1* locus, upregulated after adipogenic induction, displayed increased gene-enhancer proximity over time (Figure 3d–f). Not all genes, however, displayed significant variations in gene-enhancer distances at the nuclear periphery despite expression changes, as exemplified by the *LRATD1* locus (Figure 3g–i). 

These results provide evidence of enhancer connectivity at the nuclear periphery. They also suggest that the chromatin looping connecting genes and regulatory elements at the NL are plausible and may provide a means of regulation of gene expression in LADs.

### 3.3. Predominance of Enhancer Connections within cLADs

Concordant with earlier findings [16], we previously found that enhancers from the GeneHancer database (double elite set) [45] imputed to genes in LADs are mainly short-range enhancers [10]. To examine this further, we determined the proportions of ECHi-C cLAD bait connections which are localized within or outside LADs.

**Figure 4:**
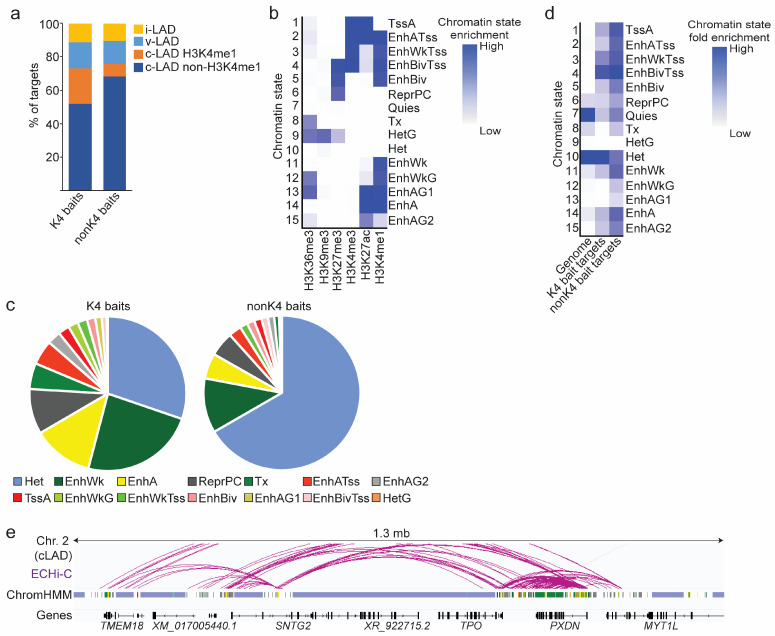
LAD and chromatin landscape of ECHi-C connections in cLADs. (**a**) Proportions of K4 and nonK4 bait targets in H3K4me1 peaks and non-H3K4me1 regions in cLADs, vLADs and inter-LADs (i-LAD). LAD data are from [10]. (**b**) Annotated 15-chromatin state model learned from ChromHMM in ASCs. TssA, active TSS; EnhATss, active enhancer at TSS; EnhWkTss, weak enhancer at TSS; EnhBivTss, bivalent enhancer at TSS; EnhBiv, bivalent enhancer; ReprPc, repressed by Polycomb; Quies, quiescent; Tx, transcription; HetG, heterochromatin in gene; Het, heterochromatin; EnhWk, weak enhancer; EnhWkG, weak enhancer in gene; EnhAG1, active enhancer in gene, type 1; EnhA, active enhancer; EnhAG2, active enhancer in gene, type 2 (weaker than EnhAG1). (**c**) Proportions of K4 and nonK4 bait targets in indicated non-Quiescent chromatin states. States with percentages below 0.3% are not shown (K4 baits: HetG; nonK4 baits: EnhWkG, EnhAG1, HetG). (**d**) Chromatin state enrichment of K4 and nonK4 bait targets determined by ChromHMM in relation to the genomic enrichment of each chromatin state. (**e**) Genome browser view of chromatin states from the 15-state ChromHMM underlying enhancer connections in the same region as in Figure 2e. Color coding of states is as in (**c**).

Data are shown in Figure 4a. (i) Over 70% of K4 bait targets were within cLADs, with about one-third of these at an H3K4me1 site. (ii) More than 20% of K4 and nonK4 baits interacted with targets in vLADs or in inter-LADs, which is consistent with our predictions of gene-enhancer contacts [10]. (iii) The majority of nonK4 baits also targeted sites within cLADs, with only a minor fraction targeting an H3K4me1 site. (iv) Lastly, most K4 and nonK4 baits targeted sites outside H3K4me1, largely in domains of H3K9me3-marked heterochromatin (see also Figure 2e). We conclude that most baited enhancers in cLADs form intra-cLAD interactions and, among these, K4 baits contact weak or strong enhancers more frequently than non-K4 baits. This suggests that enhancer-enhancer connections can occur within LADs, either within or between active regions in these LADs.

### 3.4. Chromatin Landscape of ECHi-C Connections

We next characterized the chromatin landscape of these baited enhancer targets. To this end, we initially applied the 25-state ENCODE ChromHMM [46] to ASCs, but found the ENCODE “Quiescent” state (devoid of any detectable mark) to be effectively partly enriched in H3K9me3 in our ASC dataset [28]. Thus, we profiled by ChIP-seq H3K4me1, H3K4me3, H3K27me3, H3K27ac, H3K9me3 and H3K36me3 in ASCs, and using ChromHMM, learned a 15-chromatin state model recapitulating the chromatin landscape of ASCs (Figure 4b). We then determined the distribution of cLAD baited enhancer targets across all 15 chromatin states, considering ≥ 1 bp overlap between a target with a given state to attribute a target to that state.

Among non-Quiescent states (Figure 4c), we find that K4 baits connected to (i) primarily weak or strong/active enhancer sites (60% of connections), (ii) Polycomb-repressed H3K27me3 chromatin (ReprPc; 9.5% of connections) and (iii) H3K9me3 heterochromatin (Het; 30% of connections). In contrast, most connections from nonK4 baits resided in H3K9me3 heterochromatin (Figure 4c, Het; 66.5% of connections), while 5% were found in H3K27me3 heterochromatin (ReprPc) and 28% were in enhancer regions (Figure 4c). Normalization of these connections to account for genome coverage of each state confirms the enrichment of K4 bait targets with other enhancer sites, while nonK4 baits mainly contacted silent heterochromatin (Figure 4d,e).

### 3.5. Lamin A/C Restricts Gene Expression in LADs

**Figure 5:**
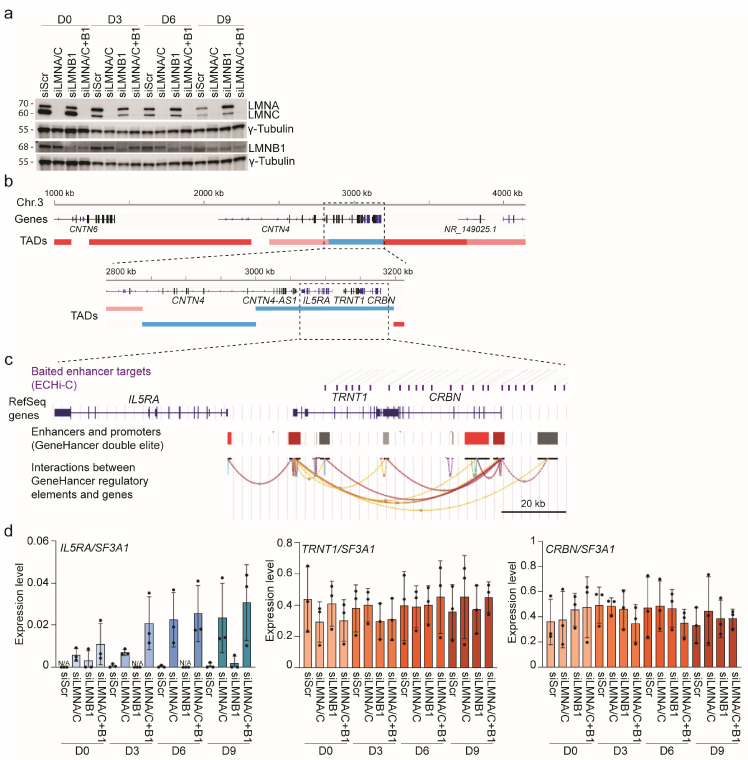
Downregulation of LMNA/C leads to the activation of a silent gene. (**a**) Immunoblotting analysis of LMNA/C and LMNB1 knock-down in ASCs; see also Figure S2. Molecular weights in KDa are shown. (**b**) Genome browser view of TADs in the *CNTN4*-*CRBN* region. The TAD track is from our earlier work and color-coded based on the size of the clique the TADs belonging to [28]: blue, singleton TADs not in clique; pink, TAD in a clique of 4 TADs; red, TAD in a clique of 6 TADs [28]. One bar in the zoom-in view represents one TAD. The zoom-in highlights the *IL5RA-TRNT1-CRBN* TAD. (**c**) UCSC genome browser view of enhancer connections in the expressed *TRNT1-CRBN* locus in an active cLAD region, along with the repressed flanking *IL5RA* gene. Enhancer connections are from ECHi-C K4 bait targets (top) and from the GeneHancer double elite enhancer set (https://www.genecards.org; accessed on 19 January 2023) [45]. (**d**) RT-qPCR analysis of *IL5RA*, *TRNT1* and *CRBN* after knock-down of LMNA/C, LMNB1 or both, and in scrambled siRNA control (siScr) cells at indicated differentiation time-points; mean ± SD of 3 experiments; individual values are shown. Expression of *CNTN4* was undetected in each experiment (not shown).

Elevated LMNB1 and LMNA/C levels flanking active cLAD regions could restrict conformational changes of chromatin [47] and hence would contribute to gene repression in these regions. To test this, we knocked down using siRNAs, LMNA/C, LMNB1 or both in undifferentiated ASCs (Figure 5a and Figure S2) and induced differentiation. We assessed by RT-qPCR expression of the repressed *IL5RA* gene, which lies immediately 5′ of the *TRNT1*-*CRBN* active region in cLAD of chromosome 3. (Figure 5b). Interestingly, IL5RA is in the same topologically associated domain (TAD) as *TRNT1* and *CRBN* in ASCs [28] (Figure 5b) and shares enhancers with these genes as part of an enhancer hub (Figure 5c). 

Whereas the expression of *TRNT1* and *CRBN* was maintained in knocked down cells, *IL5RA* expression was induced by as early as D3 after the knock-down of LMNA/C, or of both LMNA/C and LMNB1, but not after the knock-down of LMNB1 alone (Figure 5d). *IL5RA* expression increased by D6 and reached a similar level whether only LMNA/C or LMNA/C and LMNB1 were knocked down. In contrast, the neighboring *CNTN4* gene, located in a separate TAD part of a larger assembly of TADs forming a repressive TAD clique at the NL [28] (Figure 5b), was not activated after LMNA/C or LMNB1 knock-down (not shown). Thus, downregulation of LMNA/C is sufficient to elicit activation of the silent *IL5RA* locus within the *TRNT1*-*CRBN* TAD, but in the neighboring TAD. 

## 4. Discussion

We report the localization of >200 active genes in cLADs defined across early adipose differentiation time-points within euchromatic regions of low-contact frequencies with LMNB1 and LMNA/C. Of note, recent work has shown that RNA-seq underestimates the total number of effectively active (transcribing) genes at the NL, as shown by mapping nascent transcripts using global run-on sequencing (GRO-seq) [16]. Thus, transcriptional activity in LADs is expectedly to be higher than what is reported here. Lower lamin enrichment in active LAD regions [17,18,19] implies chromatin dissociation from the NL. This may be mediated by transcription [18] or by CTCF and cohesin, which also occupy active sites in LADs [19]. 

NL detachment can extend beyond the activated gene into non-transcribed regions, non-activated neighboring genes or whole transcription units [18]. This could however depend on the chromatin context surrounding the active locus. In ASCs, the repressed *IL5RA* gene, flanking the active *TRNT1-CRBN* locus, is enriched in LMNB1 and H3K9me3. All three genes reside in the same TAD [28] but only the *TRNT1-CRBN* locus is depleted of H3K9me3 and LMNB1^low^. Thus, proximal interactions with lamins may prevent activation of entire transcription units (as TADs) at the NL, as suggested by *IL5RA* activation after the downregulation of LMNA/C. 

Several possibilities may account for the effect of LMNA/C, and not LMNB1, on the regulation of *IL5RA*, and probably of other genes, in the flanks of active regions in LADs. Of note, in our system, knock-down of LMNB1 was incomplete, so we cannot formally exclude an involvement of LMNB1 in gene regulation near these regions. (i) LMNA/C is required for proper genomic localization of H3K27me3 [48]. By displacing H3K27me3, elimination of LMNA/C may favor spreading of H3K27ac into the LAD and enable *IL5RA* activation, similar to the activation of the NL-associated *Tcrb* locus after deletion of a LAD border [23]. (ii) LMNA/C, but not LMNB1, has been shown to anchor chromatin at the nuclear envelope [49]; downregulation of LMNA/C may elicit local detachment of chromatin domains containing repressed genes from the NL, creating a permissive environment compatible with induction of *IL5RA*. (iii) LMNA/C restricts chromatin mobility in the nucleus [47], so knock-down of LMNA/C may enhance chromatin motion; this could, in turn, facilitate chromatin looping events and enhancer connectivity, as well as accessibility to the transcription machinery. (iv) We have earlier shown, using a ChIP-on-chip approach, that LMNA/C can bind promoters, an event that correlates with transcriptional inactivity of the gene [50]; LMNA/C downregulation may alleviate this local repression on the *IL5RA* promoter by mechanisms involving one of more of the aforementioned possibilities. These studies altogether suggest that local detachment of chromatin from the NL may facilitate the formation of regulatory loops at or near the NL, which could bring multiple regulatory elements close to active genes. 

Accordingly, we provide evidence of multiple connections between ECHi-C baited enhancers in LADs and other enhancers also in these LADs. In bone marrow mesenchymal stem cells, enhancers have been shown using ECHi-C to exist as distinct “communities” that can be reconfigured via changes in enhancer interactions [21]. Mapping these ECHi-C baited enhancers onto cLAD in our adipogenic system suggests that some of these networks pre-exist at the NL in the form of hubs restricted to active cLAD regions (short-range interactions) or as wider networks spanning larger genomic distances. Detachment of chromatin from the NL in these regions may facilitate the remodeling of these enhancer networks.

Enhancers of active genes in LADs have earlier been inferred based on GRO-seq data and histone modifications (H3K4me1, H3K27ac) combined with a nearest target gene model [16]. In this approach, close-range enhancers are associated with highest gene activity, irrespective of NL association; however, most enhancers of NL-associated genes are found to lack NL association [16], supporting a view of long-range interactions. This could be because NL-associated genes are expressed at a lower level than genes in the nuclear interior, and that longer gene-enhancer distances correlate with lower gene expression levels [16]. In contrast, using the double-elite enhancer set from GeneHancer enriched in H3K27ac in our adipose system, we previously found that the majority of enhancers imputed to differentially expressed genes in cLADs are in cLADs [10], corroborating our ECHi-C analysis. Our study further argues that cLAD gene enhancers are weak enhancers (with low H3K27ac), which could also account for the lower expression level of genes at the NL. 

The distinct outcomes of the two studies [10,16] may be influenced by the inference method used, the nature of the expressed LAD genes (e.g., housekeeping or cell type-specific [10,17]) and the expression level of the gene [16]. Yet, both raise the possibility of chromatin loops bringing LAD genes and enhancers in proximity at the nuclear periphery. Chromatin at the NL emerges as a dynamic compartment undergoing topological remodeling at various scales [10,18,28,51] to ensure lineage specificity of gene expression. 

## Data Availability

LMNA/C ChIP-seq data, and H3K4me1, H3K4me3, H3K9me3, H3K27me3, H3K27ac and H3K36me3 ChIP-seq data generated and used in this study are available at NCBI GEO GSE221288 (https://www.ncbi.nlm.nih.gov/geo/query/acc.cgi?acc=GSE221288; accessed on 19 January 2023). Our previous H3K4me1, H3K4me3 and H3K27ac ChIP-seq data and RNA-seq data are available at NCBI GEO GSE185066 (https://www.ncbi.nlm.nih.gov/geo/query/acc.cgi?acc=GSE185066; accessed on 19 January 2023) [10]. Our previous LMNB1 and H3K9me3 ChIP-seq are available at NCBI GEO GSE109924 (https://www.ncbi.nlm.nih.gov/geo/query/acc.cgi?acc=GSE109924; accessed on 19 January 2023) [28]. ECHi-C data are available at NCBI GEO GSE140782 [21] and were lifted to hg38. ATAC-seq data are available from NCBI GEO GSE143449 [37]. Enhancers used to design FISH probes were from the GeneHancer double-elite subset (https://www.genecards.org; accessed on 19 January 2023) [45].

## Supplementary Materials

The following supporting information can be downloaded at: https://www.mdpi.com/article/10.3390/genes14020334/s1, Figure S1: LMNA/C enrichment in the ASC genome; Figure S2: Western blot assessment of lamin knock-downs; Table S1: FISH probe information; Table S2: RT-qPCR primers; Table S3: Expressed and non-expressed genes in cLADs (Excel); Table S4: Enriched gene ontology terms for active genes in cLADs (Excel).

## Abbreviations

ASC, adipose stem cell; ATAC, assay for transposase-accessible chromatin; ChIP, chromatin immunoprecipitation; DE, differentially expressed; Hi-C, high-throughput chromosome conformation capture; ECHi-C, enhancer capture Hi-C; GEO, Gene expression omnibus; LAD, lamina-associated domain; cLAD, constitutive LAD; vLAD, variable LAD; NL, nuclear lamina; seq, sequencing.
