# Supplementary material for: Gene Regulatory Interactions at Lamina-Associated Domains
Source: Genes (Basel). 2023 Jan 28;14(2):334. doi: 10.3390/genes14020334 (PMC9957430; doi:10.3390/genes14020334)
Supplement: Supplementary file 1 [file genes-14-00334-s001.zip › Osterbye 2023 Genes SUPP.pdf]

Supplemental information

Table S1. FISH probe information.

Probe ID	Clone ID*	Chr.	Start pos.	End pos.	Gene/Enhancer
P1	WI2-2754C15	Chr.12	108.300.483	108.344.051	G: *CMKLR1*
P2	WI2-3662N9	Chr.12	108.810.063	108.851.447	E: *CMKLR1*
P3	WI2-3098N15	Chr.2	14.616.693	14.654.959	G: *LRATD1*
P4	WI2-2169G17	Chr.2	15.152.021	15.189.213	E: *LRATD1*
P5	WI2-2387C14	Chr.10	23.425.270	23.461.648	G: *OTUD1*
P6	WI2-3761J1	Chr.10	23.066.127	23.106.499	E: *OTUD1*

*BACPAC Genomics (bacpacresources.org)

Table S2. RT-qPCR primers.

Gene	Primer
SF3A1	F: AGGGTCCAGTGTCCATCAAA<br>R: AGAGACCTGGTCCGTGAGTG
TRNT1	F: GCGGGGCCGATGTGTAG<br>R: TACTGCTTCGGAAGGCACAG
CRBN	F: CCAGTCTGCCGACATCACAT<br>R: TGTCTGTCCGGGAATCAGGA
IL5RA	F: CTGTGCCTGACGCTATGCTA<br>R: ATCTCAGTGGCCCCCAAAAG

Table S3. Expressed and non-expressed genes in cLADs (Excel).

Table S4. Enriched gene ontology terms for active genes in cLADs (Excel).

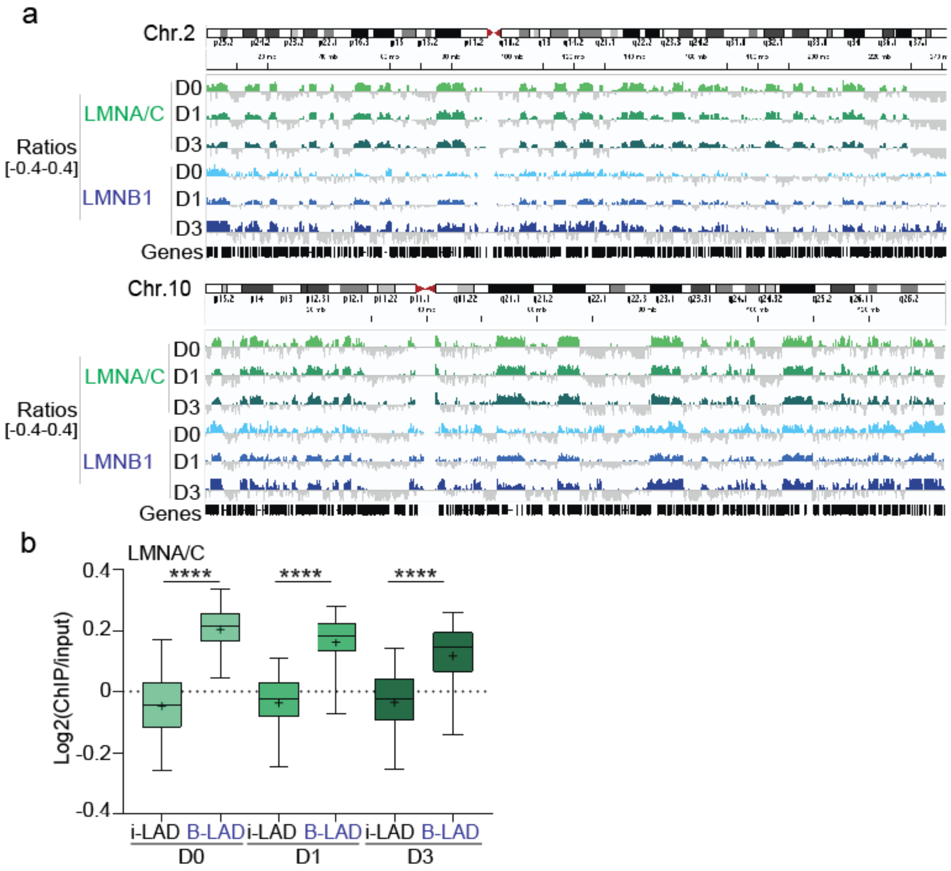

Figure S1. LMNA/C enrichment in the ASC genome. **(a)** Genome browser examples of LMNA/C enrichment as Log2(ChIP/Input) ratios determined by ChIP-seq; chromosomes 2 and 10. Ratios of LMNB1 enrichment are from our previous work [1]. **(b)** LMNA/C enrichment in LMNB1 LADs (B-LADs) and i-LADs; bar, median; cross, mean; box, 25-75% percentile; whiskers, min-max ****P < 10^{-4}, ANOVA with Welch's correction.

1. Madsen-Østerbye, J., Abdelhalim, M., Baudement, M.O., Collas, P. Local euchromatin enrichment in lamina-associated domains anticipates their re-positioning in the adipogenic lineage**.** *Genome Biol* **2022**, *23*, 91.

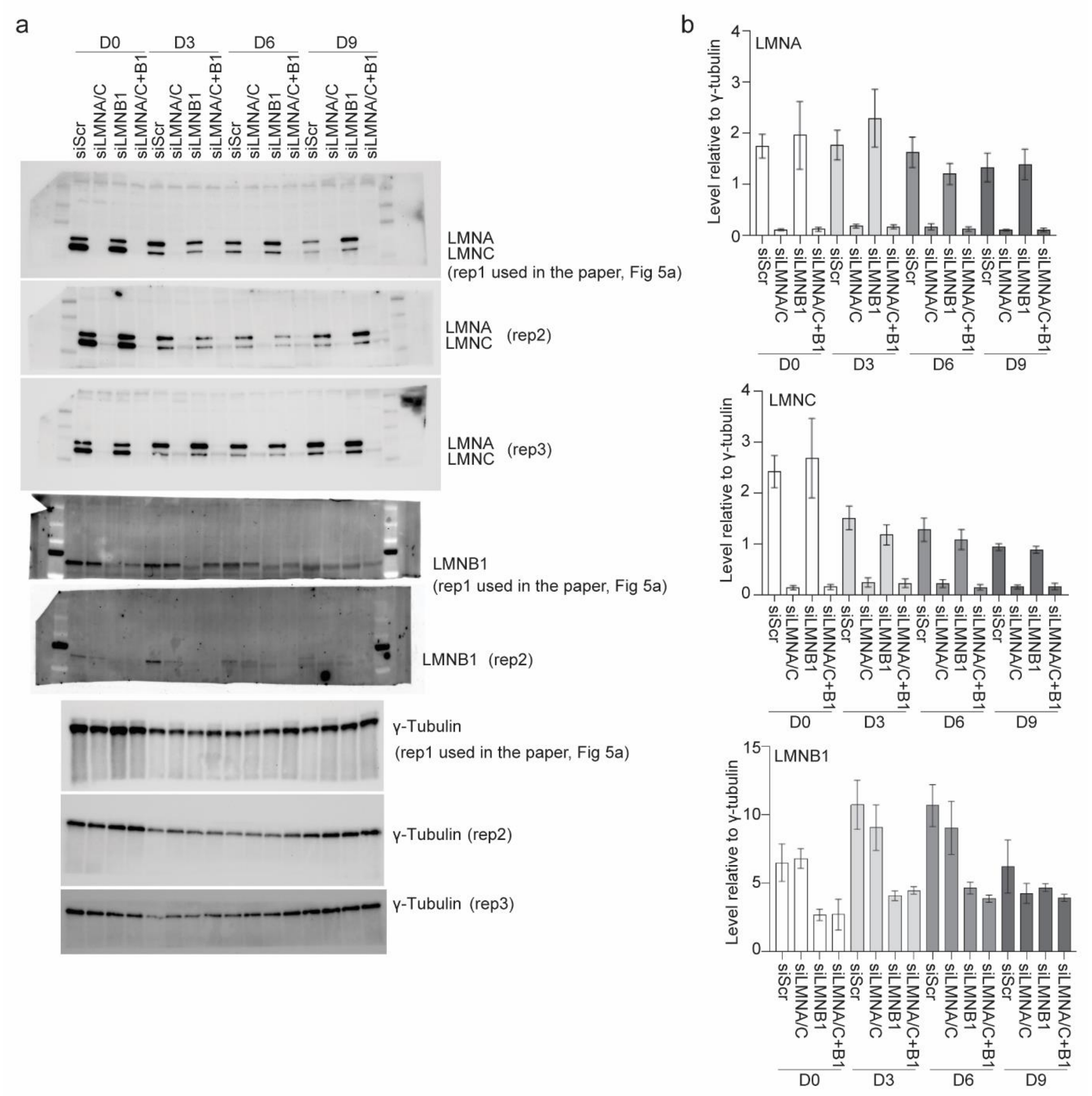

Figure S2. Western blot assessment of lamin knock-downs. **(a)** Uncropped Western blots. **(b)** Quantifications of the blots shown in **(a)**; mean ±SD; n = 3 and n= 2 experiments for LMNA/C and LMNB1 blots, respectively.
